# Characterization of a 100 nm RADFET as a Proton Beam Detector

**DOI:** 10.3390/s26010202

**Published:** 2025-12-27

**Authors:** J. A. Moreno-Pérez, I. Ruiz-García, R. Duane, P. Martín-Holgado, L. Morvaj, N. Vasovic, W. Hajdas, Y. Morilla, M. A. Carvajal

**Affiliations:** 1 PRIMELab, ECsens, Sport and Health University Research Institute (iMUDS), Department of Electronics and Computer Technology, Research Centre for Information and Communications Technologies (CITIC), University of Granada, 18071 Granada, Spain; juanantoniomp@ugr.es; 2 James Watt School of Engineering, University of Glasgow, Glasgow G12 8QQ, UK; isidoro.ruizgarcia@glasgow.ac.uk; 3 Varadis, Site 13 Carrigaline Industrial Park, Carrigaline, P43 DC53 Cork, Ireland; russell@varadis.com (R.D.); nikola@varadis.com (N.V.); 4 Centro Nacional de Aceleradores, Universidad de Sevilla, CSIC, JA, Avda. Tomás Alva Edison 7, 41092 Sevilla, Spain; pmartinholgado@us.es (P.M.-H.); ymorilla@us.es (Y.M.); 5 PSI Center for Neutron and Muon Sciences, PSI, 5232 Villigen, Switzerland; ljiljana.morvaj@psi.ch (L.M.); wojtek.hajdas@psi.ch (W.H.)

**Keywords:** radiation detector, RADFET, unbiased, proton beams, dosimetry

## Abstract

The RADFET VT06 developed by Varadis (Cork, Ireland), which is aimed at high-dose applications, mainly for spacecraft missions, has been characterized by low- and high-energy proton beams at two different facilities, the Accelerator National Centre (Sevilla, Spain) and the Paul Scherrer Institute (PSI) located in Villigen (Switzerland), using a reader unit system developed by the University of Granada (Spain). The devices have been characterized with proton energies of 1, 2, 3, 150, and 230 MeV, with accumulated doses from 130 to 512 Gy, where the RADFET was unbiased during the irradiation while the source voltage was measured before and after irradiation to monitor the radiation dose. Excellent linearity has been obtained with a minimum correlation factor R^2^ of 0.996, with a sensitivity that can vary from (0.691 ± 0.007) mV/Gy for 1 MeV to (1.143 ± 0.023) mV/Gy for 230 MeV without any build-up layer. An excellent stability was found in the studied cases, with dispersion being lower than 4% after a dose accumulation higher than 500 and 200 Gy for protons of 1 and 3 MeV, respectively. The detectors demonstrated linear responses, very low sensitivity dispersion per set of samples, and excellent stability after irradiation. This shows that, with an appropriate readout system, the RADFET can become an excellent system for high-dose proton beams.

## 1. Introduction

Ionizing radiation is characterized by the creation of ion pairs in materials. These ionic pairs cause failures in electronic circuits, which can become permanent defects, and biological damage to the DNA of human and animals, which can produce tumours and death in extreme cases. Therefore, the detection of ionizing radiation and quantification of the energy delivered by the radiation is crucial in facilities with radiation sources such as particle accelerators, nuclear plants, and hospitals with radiotherapy services, among others. Therefore, in recent decades, different types of radiation detector have been developed, such as ionization chambers, thermoluminescent dosimeters (TLDs), radiochromic films, and, especially, semiconductor detectors [1,2,3]. Semiconductor devices such as diodes or metal-oxide field effect transistors (MOSFETs) [4,5,6,7] have been used as radiation detectors and dosimeters due to their low size, high reproducibility, and immediate readout among other characteristics. In the case of MOSFETs, the ionizing radiation produces electron–hole pairs, which are separated both in the gate oxide and at the Si-SiO_2_ interface, producing an increment of the threshold voltage (V_t_) [8,9]. So, to improve the sensitivity, a thicker gate oxide is grown using a special manufacturing process, and these transistors are known as RADFETs [10,11,12]. The main disadvantage of a thick gate oxide is an increase in the threshold voltage. To reduce the V_t_, some techniques, like ionic implantation, are used. In recent years, RADFETs have been investigated using an insulator material between the gate and the bulk with a high dielectric constant, which are called high-K transistors and which can provide a high sensitivity with a thin dielectric layer [13,14,15].

The sensitivity of a MOSFET dosimeter mainly depends on the gate oxide characteristic and the applied electric field. The internal electric field in the gate dielectric can be increased by applying an external voltage during irradiation [12,16,17], and in this case, the transistor is operating in biassed mode. A MOSFET can accumulate charge in the gate-oxide even without external voltage during irradiation due to in-built electric field in the dielectric. It is sufficient to short-circuit all the terminals, and then the transistor is operating as a dosimeter in unbiased mode. However, the induced charge does not remain in the gate oxide indefinitely as the net charge can suffer recombination after irradiation, reducing the threshold voltage shift. This is the fading effect, which depends strongly on temperature [17,18,19].

Threshold voltage shifts can be calculated by measuring the current–voltage curve before and after irradiation using a semiconductor analyser. However, the most usual technique is to measure the source voltage with a constant drain current implemented in a reader unit [16,20,21].

In the case of high-dose applications, like environmental monitoring in radioactive facilities or spacecraft missions [22], robust devices should be used. In this sense, some companies have developed devices like the RADFET VT06 from Varadis (Cork, Ireland), which operates in the dose range from 10 Gy to 10 kGy [23]. This device is focused on use as a dosimeter in space applications, where the systems receive a wide energy spectrum of ionizing radiation, especially high-energy particles like photons, electrons, or protons. Therefore, a wide characterization with different types of ionization radiation is advisable.

Previously, the response of some RADFETs has been studied under proton beams at different energies. For example, the RADFET ESAPMOS4 design manufactured at Tyndall National Institute with a gate oxide of 400 nm was characterized as detector of 60 MeV proton beams with different biassing configurations [24]; the RADFET TN502RD, manufactured by Best Medical (Springfield, VA, USA) was tested as dosimeter for proton beams as well, but with energies of 190 MeV, studying the sensitivity and angular dependence [25]. In both cases, the device under test presented reliable characteristics for use as a dosimeter with proton beams.

In the current work, the characterization of the dosimeter VT06 under low- and high-energy proton beams is presented. We have reconfigured our reader system [16] to operate with this RADFET using an experimental setup that was validated for measurements with low-energy protons in the characterization of a general purpose MOSFET as a dosimeter [26].

## 2. Materials and Methods

The device that was characterized was the RADFET VT06 manufactured by Varadis (Cork, Ireland). This is a pMOS transistor with a gate oxide thickness of 100 nm aimed at radiation measurements in aerospace applications.

**Figure 1:**
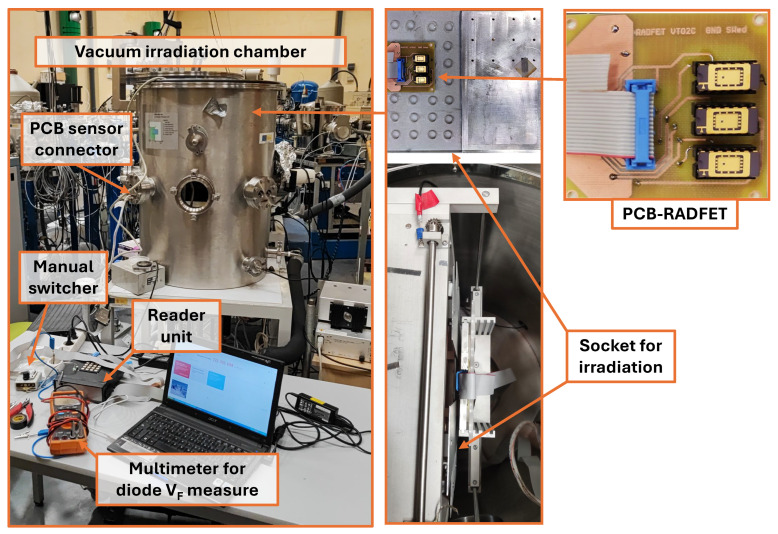
Experimental setup for low-energy proton irradiations in the Tandem accelerator (CAN, Sevilla, Spain).

In the current work, a characterization at low and high energies was carried out under proton beams. The irradiation tests at low energies were conducted in the National Centre of Accelerator (CNA) in Sevilla (Spain) using the Tandem Accelerator for generating proton beams of 1, 2, and 3 MeV. Due to the low penetration power of low-energy proton beams, the samples were placed in a vacuum chamber through which the proton beam reached the devices under test. The top of the housing was also removed, as shown in Figure 1. To characterize performance under high-energy proton beams, the RADFETs were irradiated in the Proton Irradiation Facility (PIF) in the Paul Scherrer Institute (PSI) located in Villigen (Switzerland) for typical energies of 150 and 230 MeV, using a similar experimental setup but with some important differences, which will be described later.

**Figure 2:**
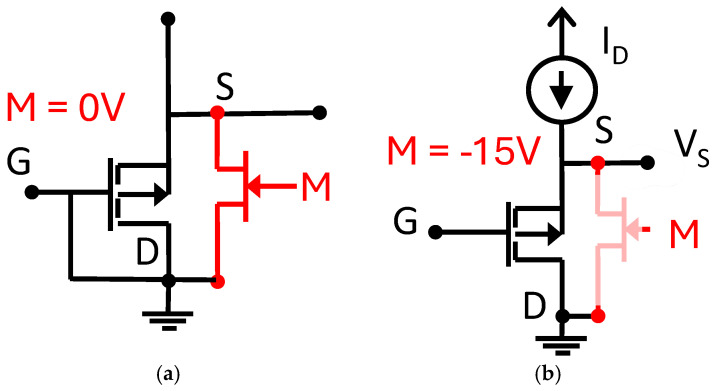
Schema of the JFET (in red colour) and RAFET (in black) during (**a**) irradiation and storage state and (**b**) readout state.

The RADFETs were placed on a printed circuit board (PCB) connected to a JFET (model MMBF4391 NXP Semiconductors, Eindhoven, The Netherlands) by a pMOS transistor, as Figure 2 shows. During the storage periods and during irradiation, when the PCB is disconnected from the reader unit, the gate of the JFET is at the same voltage as the ground through the pull-down resistor (100 kΩ in our case). Therefore, the JEFT connects the S-B (source and bulk terminals) and the G-D (gate and drain terminals) as Figure 2a shows. Before and after irradiations, the transistor is connected to the reader unit where the gate of the JFET is biassed at −10 V, breaking the connection between the S-B and the G-D terminals (see Figure 2b). Then, the readout process is carried out, and the current flows through the RADFET, as Figure 2 shows, where the source voltage is registered and stored in the non-volatile memory of the reader. The source voltage (V_S_) was registered before and after irradiations and downloaded directly to the PC. The internal diode included in the RADFET silicon die was used to monitor the temperature of the devices through its forward voltage (V_F_). The drain current was provided by the reader, and the source voltage was registered. To improve resolution and the signal-to-noise ratio (SNR), the amplified channel of the reader unit was used, which involves an amplification based on instrumentation amplifier and a digital-to-analogue converter (DAC). The output of the DAC is connected to the inverting input of an instrumentation amplifier, and the buffered source voltage is connected to the non-inverting input. During the zeroing process, the DAC is set to force the output of the instrumentation amplifier at a low positive voltage [16]. To minimize the temperature dependence, the drain current of the transistors was set at I_ZTC_ (Zero Temperature Coefficient), which, for this model of RADFET, is equal to 17 µA [27].

To manage six transistors per irradiation session, a manual switcher was developed ad hoc to connect the transistors one by one. Two measurement strategies were applied: the continuous mode, measuring the source voltage continuously [16], and the difference mode [26], measuring the source voltage at the beginning and the end of the irradiation. This process starts with the zeroing process before irradiation and finishes measuring the source voltage shift at least two minutes later after irradiation stops to ensure the short-term fading of the transistors was finished [16]. In more detail, the measurement process for the difference mode can be summarized as follows:

1. Zeroing: The initial source voltage is recorded per each transistor (switching manually).
2. Irradiation: The devices are irradiated with all terminal short-circuited.
3. Measurement: After at least two minutes since the radiation stops, the source voltage shift is measured and stored per transistor.
4. Temperature monitoring: The forward voltage of the chip diode is registered.

In the case of continuous mode, after the zeroing, the system continues measuring the source voltage until some minutes after the end of the irradiation.

The RADFET PCB was placed on one specific sample holder to be used in the vacuum chamber (Figure 1). The PCB was designed to contain three chips (with two devices per chip), which means six samples were irradiated simultaneously per session. The beam was scanned 10 cm × 10 cm to cover the samples simultaneously during each run. The total fluence is calculated based on the value of the particle density integrated into the complete area. During the focalization process of the beam, only the scintillator (EJ-440 ZnS(Ag) phosphor sheet) was exposed, and the samples remained masked behind the slits. The scintillator was not used as a dosimeter but rather to ensure that the beam reached this position and the produced light was acquired by a digital camera and displayed the operator´s room remotely. The reader unit was managed directly close to the vacuum chamber, as Figure 1 shows. This experimental setup was validated in a previous work [26] for the characterization of a modified commercial transistor as a low-energy proton detector.

**Figure 3:**
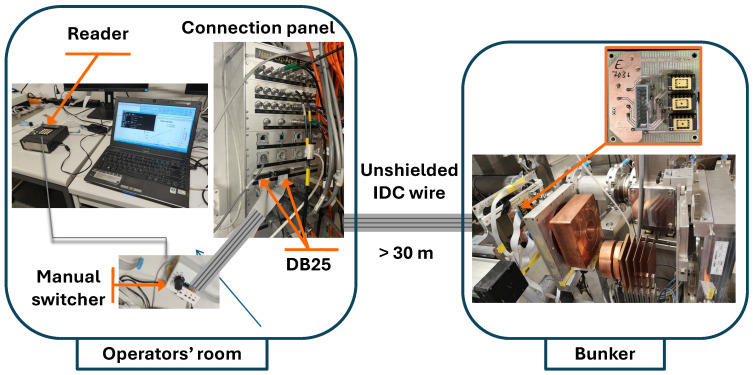
Schematic of the experimental setup in PIF for high-energy proton beam measurements.

For high-energy proton beams, the RADFETs were irradiated in the Proton Irradiation Facility with energies of 230 and 151.53 MeV (nominal value of 150 MeV). The experimental setup was very similar to the configuration used for low-energy protons at the CNA (Sevilla, Spain) but with an important difference, and it is that the PIF was placed in an area with a medium level of radiation; therefore, the researchers had to stay far away from this area, in the operator’s room, which was located more than 30 m from the bunker. An unshielded cable connects two identical connector panels; one of them is located in the bunker and the other in the operator’s room. We placed an ad hoc adapter from DB25 to the manual switcher in the operator’s room, as Figure 3 shows, and another identical one connected the DB25 panel connector with the PCB-RADFET in the bunker. Due to the long distance from the samples in the bunker to the reader unit in the operator’s room and also due to the unshielded wires and connectors, the noise level was much greater than that observed in the CNA conditions.

The number of samples studied in every irradiation test depended on the availability of space in the irradiation area and beam time. No restrictions were found in CNA tests regarding the space and time; therefore, six samples were irradiated for every energy, and one of them were monitored continuously to check that the proton beam reached the transistors with an accumulated dose higher than 200 Gy. The available irradiation time in PIF was shorter than in CNA, and therefore only two samples were characterized per energy. This reduction in samples was also due to the fact that the surface in the irradiation area was shared with other characterization experiments, and the maximum dose that was provided was 130 Gy. The total accumulated doses achieved in each irradiation session are summarized in Table 1, and in the last row, the number of samples characterized in difference mode plus the samples that were monitored continuously are recorded. In the case of the 1 MeV study, a final long run was conducted to study the recovery of the source voltage at higher doses.

## 3. Results and Discussion

**Figure 4:**
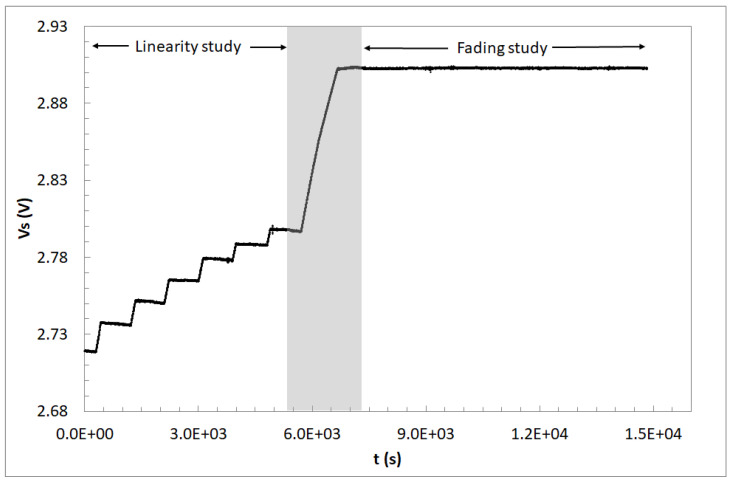
Source voltage of the sample #1.6 during 1 MeV irradiations in CNA.

In order to study the sensitivity response, a series of irradiation runs were conducted with a variable dose depending on the energy, achieving a minimum total dose varying from 130 Gy to 218 Gy. To study the recovery of the signal after irradiation, an additional run (number 7 in Table 1) was conducted to achieve a total dose of 552.1 Gy. To ensure that the proton beam reached the samples, one of the transistors was monitored continuously during the low-energy proton beam experiments, as is detailed in Table 1. Samples that were measured continuously were not considered in the linearity and sensitivity studies in difference mode due to the lower sensitivity in continuous mode. During the readout configuration, the electric field in the gate oxide, which is the detection volume, is negative, and this negative electric field reduces the sensitivity of the RADFET if exposed to radiation during readout. As a result, a lower sensitivity is found if the transistor is continuously monitored. The samples were irradiated according to Table 1. In Figure 4, the source voltage during the irradiation test and post-irradiation drift is plotted. The first six runs are the irradiations corresponding to the sensitivity study, while a final longer irradiation was provided to produce a wider V_S_ shift. Therefore, a run of 334.1 Gy was carried out to start the recovery study with a high value of V_S_. The short-term fading was studied in the following minutes using the continuous mode, and the long-term fading was studied with the rest of the samples of the set irradiated with 1 MeV using difference measurement mode.

### 3.1. Sensitivity and Linearity

**Figure 5:**
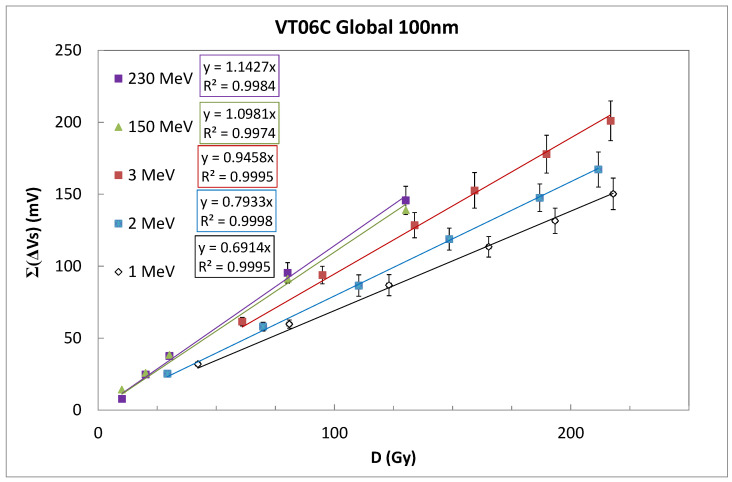
Accumulated average source voltage per set of transistors as function of the total dose absorbed at different energies.

The accumulated source voltage shifts were calculated in the irradiation test using six irradiations for the low-energy proton beam (achieving a total dose of 210 to 220 Gy) and five irradiation sessions for the high-energy proton beam (up to 130 Gy). The global value of the increment per irradiation session was calculated as the average of the shift in each individual transistor, and the uncertainty was found as the square propagation error of the experimental error and standard deviation of the set of transistors. Sensitivity was calculated as the slope of the linear regression of accumulated voltage shifts as a function of the accumulated dose (see Figure 5), and linearity was evaluated using the correlation factor (R^2^) of this linear regression.

The transistors exhibited an excellent linearity and low dispersion among the samples, which implies reduced error bars, as can be seen in Figure 5. The sensitivities found per sample for the different energies are summarized in Table 2. The average of the individual sensitivities per sample was inputted into each set of RADFETs discarding the sample used in continuous mode, represented by Avg. in Table 2. The global sensitivity value was calculated as the slope of the linear fit using the average of the individual of source voltage shifts per irradiation session (Figure 4).

### 3.2. Fading

The sensitivity change with proton energy is interesting and most pronounced for 1 MeV, 2 MeV, and 3 MeV. It may be that the sensitivity is reduced in this energy range due to the protons being shielded partially by the aluminum gate, as other authors found studying the effects of low-energy protons in NAND flash memories [28].

The source voltage recovery was studied during the following hours after the last irradiation of 1 and 3 MeV when the transistors had accumulated 552.1 and 216.9 Gy, respectively. At high energies, the samples became radioactive due to the activation produced by the protons, and therefore it was not possible to study the fading. For low energies, two strategies were used to evaluate the decay of the source voltage: one sample was continuously monitored, measuring the source voltage every two seconds, and, for the other five samples, the source voltage was measured once after fifteen hours.

**Figure 6:**
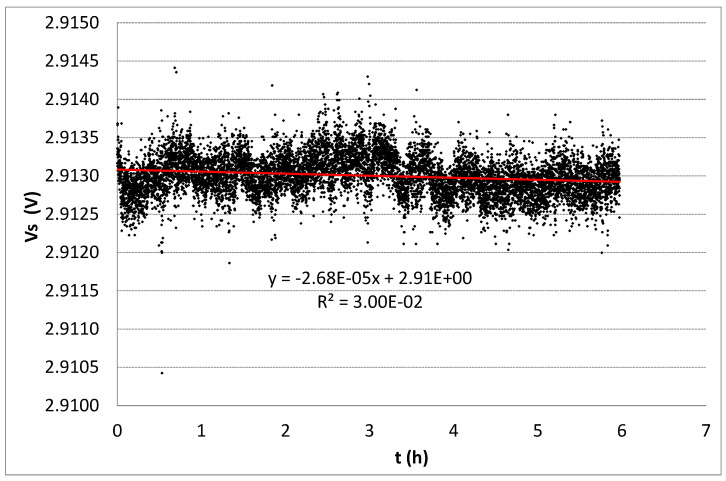
Source voltage drift measured every two seconds for sample #1.6 after irradiation with 1 MeV proton beam up to 552.1 Gy (Si).

In continuous mode, the source voltage for one sample per energy was monitored continuously during six hours for 1 and 3 MeV. In Figure 6, the drift of sample #1.6 is plotted, showing a low decay (−0.0268 ± 0.0015) mV/h, while for sample #3.6, irradiated with 3 MeV, a decay of (−0.052 ± 0.020) mV/h was found. Taking into consideration the sensitivity, these values imply a drift of (−7.2 ± 0.4) cGy/h for 1 MeV and (−13.7 ± 0.5) cGy/h for 3 MeV. Even with a continuous measurement process, the source voltage remained stable, demonstrating an excellent stability, although a huge dose was absorbed.

**Figure 7:**
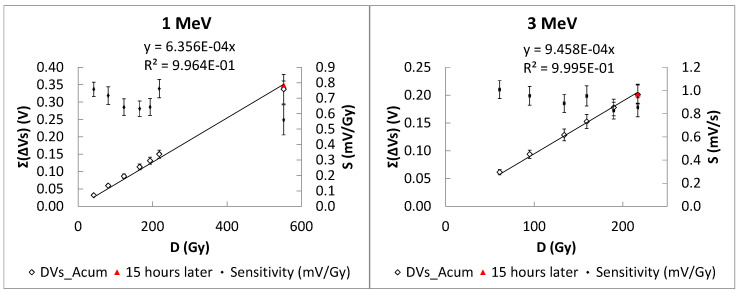
Accumulated source voltage shift during irradiations (in white), sensitivity (in black), and the value of the accumulated source voltage after fifteen hours for 1 and 3 MeV.

In the difference mode, a unique measurement of the source voltage was carried out after fifteen hours. The source voltage grew in average (five samples) by a total of (6.3 ± 0.9) mV after an accumulated increment of (201 ± 17) mV due to a corresponding absorbed dose of 216.9 Gy. This fading represents 3.1% of the total increment for 3 MeV (see Figure 7). In the case of 1 MeV, the total dose was 552.1 Gy, which produced a voltage shift of (337 ± 43) mV, and after fifteen hours, the source voltage increased by (12 ± 23) mV, which represents 3.6% of the total increment.

## 4. Conclusions

A characterization of the RADFET VT06 from Varadis has been carried out for low- and high-energy proton beams (1, 2, 3, 150, and 230 MeV). A set of six transistors in unbiased mode have been tested per energy using a reader unit developed at the University of Granada. The results show very low dispersion among the samples per set, even in high-proton energy measurements where the reader was located more than 30 m from the samples.

The sensitivities found per each set at energies of 1, 2, 3, 150, and 230 MeV was (0.691± 0.007) mV/Gy, (0.793 ± 0.005) mV/Gy, (0.946 ± 0.009) mV/Gy, (1.09811 ± 0.00003) mV/Gy, and (1.143 ± 0.023) mV/Gy, respectively, with very high linearity (R^2 higher than 0.996). In addition, the recovery of the source voltage, after fifteen hours, was about 3% after 500 and 200 Gy for 1 and 3 MeV.

These characteristics have demonstrated that the VT06 is a very suitable device to monitor high doses under low- and high-energy proton beams.

## Figures and Tables

**Table 1:** Total accumulated dose, in Gy (Si), achieved with the different irradiation sessions. Two last rows: devices under test (DUTs) irradiated in difference mode (D.M.) and in continuous mode (C.M.).

	CNA			PIF	
Run	1 MeV	2 MeV	3 MeV	150 MeV	230 MeV
1	42.2	29.3	61.0	10.03	10.08
2	80.9	69.8	94.9	20.09	20.09
3	123.1	110.3	133.9	30.16	30.14
4	165.3	148.6	159.3	80.24	80.19
5	193.4	186.9	189.8	130.24	130.2
6	218.0	211.7	216.9	--	--
7	552.1	--	--	--	--
DUTs in D.M.	#1.1,…,#1.5	#2.1,...,#2.5	#3.1,...,#3.5	#150.1, #150.2	#230.1, #230.2
DUTs in C.M.	#1.6	#2.6	#3.6	--	--

**Table 2:** Sensitivity per transistor, average and global values in difference mode, and, in grey, the sensitivity in continuous mode, excluded in the study of difference mode.

	Sample	S (mV/Gy)	ΔS (mV/Gy)	R^2^		Sample	S (mV/Gy)	ΔS (mV/Gy)	R^2^
1 MeV	#1.1	0.729	0.006	0.9996	3 MeV	#3.1	1.038	0.010	0.9995
	#1.2	0.633	0.007	0.9994		#3.2	0.868	0.010	0.9994
	#1.3	0.721	0.006	0.9997		#3.3	0.967	0.009	0.9996
	#1.4	0.637	0.008	0.9993		#3.4	0.860	0.009	0.9994
	#1.5	0.737	0.007	0.9995		#3.5	0.996	0.008	0.9997
	#1.6	0.375	0.003	0.9996		#3.6	0.568	0.009	0.9989
	Avg	0.69	0.05	--		Avg	0.95	0.08	--
	Global	0.691	0.007	0.9995		Global	0.946	0.009	0.9995
2 MeV	#2.1	0.865	0.005	0.9998	150 MeV	#150.1	1.109	0.023	0.9983
	#2.2	0.738	0.005	0.9998		#150.2	1.09	0.03	0.9960
	#2.3	0.824	0.005	0.9998		Avg	1.10	0.03	--
	#2.4	0.732	0.005	0.9998		Global	1.10	0.03	0.9974
	#2.5	0.807	0.005	0.9998	230 MeV	#230.1	1.20	0.03	0.9981
	#2.6	0.596	0.038	0.9804		#230.2	1.089	0.021	0.9985
	Avg	0.79	0.06	--		Avg	1.14	0.02	--
	Global	0.793	0.005	0.9998		Global	1.143	0.023	0.9984

## Data Availability

Data will be made available on request.

## Abbreviations

The following abbreviations are used in this manuscript:

MOSFET	Metal Oxide Field Effect Transistor
RADFET	RADiation FET
CNA	Accelerator National Centre
PSI	Paul Scherrer Institute
PIF	Proton Irradiation Facility
